# Trends in attitudes towards gambling among Finnish women and men: Cross-sectional population studies 2011, 2015, 2019 and 2023

**DOI:** 10.1177/14550725251320729

**Published:** 2025-03-12

**Authors:** Tanja Grönroos, Heli Hagfors, Jukka Kontto, Tiina A. Latvala, Anne H. Salonen

**Affiliations:** Department of Health Services, 3837 Finnish Institute for Health and Welfare, Helsinki, Finland; Faculty of Social Sciences, 7840 Tampere University, Tampere, Finland; Department of Public Health, 3837 Finnish Institute for Health and Welfare, Helsinki, Finland; Department of Health Services, 3837 Finnish Institute for Health and Welfare, Helsinki, Finland; Department of Health Services, 3837 Finnish Institute for Health and Welfare, Helsinki, Finland; Faculty of Social Sciences, 3835 University of Helsinki, Helsinki, Finland; Faculty of Health Sciences, University of Eastern Finland, Kuopio, Finland

**Keywords:** Attitudes, cross-sectional, gambling, gender, population study

## Abstract

**Aims:** To compare the attitudes towards gambling in Finland by age among women and men from 2011 to 2023. **Methods:** Four cross-sectional random sample data sets of 15–74-year-olds were collected in 2011, 2015, 2019 and 2023. The data were weighted based on gender, age and residential area. The eight-item Attitude Towards Gambling Scale (ATGS-8) was used. **Results:** Overall, public attitudes towards gambling were generally unfavourable each year, apart from 2015, when attitudes were favourable. Among women, attitudes became more unfavourable for those aged 15–29 years, whereas they became more favourable for those aged 45 to 74. Among men, attitudes became more unfavourable for those aged 15–44 years, whereas they became more favourable for those aged 60–74 years. Men aged 45–59 years were the only age group with favourable attitudes in 2023. **Conclusions:** Attitudes towards gambling were mainly unfavourable, with no notable shift observed between 2011 and 2023. The Finnish gambling monopoly system is set to be replaced by a licensing system, opening the online gambling market to competition. Because of this change, it is increasingly important to monitor attitudes towards gambling and possible changes in them in the coming years.

## Introduction

Attitudes towards gambling are linked with both gambling and gambling problems, and vice versa (Canale et al., 2016; Kristensen et al., 2023). In the Nordic context, past year gambling prevalence is highest in Finland (THL, 2024; Folkhälsomyndigheten, 2023; Pallesen et al., 2023; Spillemyndigheden, 2022; Ólason, 2024), although gambling prevalence has decreased based on the most recent population survey (THL, 2024). Furthermore, in the Nordic context, at-risk and problem gambling prevalence is the highest in Finland and in Norway (THL, 2024; Pallesen et al., 2023; Folkhälsomyndigheten, 2023; Spillemyndigheden, 2022; Ólason, 2024). Information on public attitudes towards gambling can provide essential guidance for governments as they seek policies and methods to prevent and reduce gambling-related harm (Latvala et al., 2019; McAllister, 2014). Understanding attitudes is crucial because they can shape the direction of regulation, political decisions and gambling reforms. More favorable attitudes towards gambling may suggest greater liberalization of gambling, whereas negative attitudes are often cited to justify stricter regulations on the industry (Delfabbro & King, 2020). In the near future, Finland's gambling monopoly is set to be replaced by a licensing system, opening the online gambling market to competition.

The Attitudes Towards Gambling Scale (ATGS) is often used to assess attitudes towards gambling. The ATGS was originally developed for the 2007 British Gambling Prevalence Survey (Wardle et al., 2007, 2011; Orford et al., 2009). This 14-item scale was the first standardized tool to measure gambling attitudes in a large-scale epidemiological survey (Wardle et al., 2007). Subsequently, a shortened eight-item version, the ATGS-8, was developed (Wardle et al., 2011). According to a systematic literature review published in 2023, a total of 26 papers reported 23 unique studies utilizing the ATGS across several different countries (Kristensen et al., 2023). Subsequent to the review being published, at least one peer-reviewed article has appeared, where several other opinion-related predictors of attitudes were identified (Macey et al., 2023). Furthermore, recent Nordic population-based national gambling surveys have reported the results with the ATGS in their basic reports (Pallesen et al., 2023; THL, 2024; Ólason, 2024).

Internationally, public attitudes towards gambling have been generally unfavourable or neutral, and they have mainly shifted towards even more unfavourable (Kristensen et al., 2023; Dowling et al., 2021). Similarly, gambling participation has shown a downward trend, whereas online gambling has increased in several western countries (Salonen & Hagfors, 2020). Internationally, attitudes towards gambling differ based on gender and age. Generally, women tend to have more unfavourable attitudes than men (Kristensen et al., 2023; THL, 2024). However, the link between attitudes and gender is not totally clear because some studies have reported opposite findings (Dowling et al., 2021) or found no statistically significant differences between men and women (Ayandele et al., 2021; Àndra et al., 2022). Similarly, previous studies that have investigated the relationship between attitudes towards gambling and age have produced contradictory findings: some reported a positive association between age and favourable attitudes (Pallesen et al., 2016, 2020), whereas others found no statistically significant link (Donaldson et al., 2016; Gavriel-Fried, 2015; McAllister, 2014). However, in most studies, younger individuals have reported generally more favourable attitudes than older ones (Kristensen et al., 2023).

Despite the fact that attitudes towards gambling have been studied by gender and age, studies within gender are rather scarce. Women and men differ not only in their attitudes towards gambling, but also in their motivations to gamble, gambling behaviours and the development of gambling problems (Salonen et al., 2017; Abbott et al., 2018; Pallesen et al., 2020). These findings highlight the need for further research on attitudes towards gambling separately in women and men. Attitudes towards gambling play a key role in shaping the choice to engage in gambling and the development of problem gambling (Williams et al., 2012). According to the theory of planned behaviour (Ajzen, 1991; 2012), attitudes and behaviour are closely linked. The theory proposes that behaviour can be predicted by individual's attitudes towards the behaviour, subjective norms concerning the behaviour and perceived control over the behaviour (Martin et al., 2010; Sussman & Gifford, 2019). That is, a positive attitude towards the behaviour increases the likelihood of engaging in it. Therefore, understanding these attitudes is crucial when devising prevention strategies (Velasco et al., 2021). Thus, the present study aimed to examine the following questions: (1) how attitudes towards gambling differ between age groups and within gender, and (2) how these attitudes have shifted from 2011 to 2023. To do so, the study seeks to fill the gap in understanding the demographic-specific changes in gambling attitudes over time, providing insights into how different age groups within each gender perceive and respond to gambling-related issues. This analysis will help to identify trends and patterns in attitudes, which could inform more targeted and effective policy interventions for reducing gambling-related harms. This article describes the situation and trends before the change in the Finnish gambling monopoly system.

## Methods

### Participants and measures

Data from four nationally representative cross-sectional Finnish Gambling population studies conducted in 2011, 2015, 2019 and 2023 were used (THL, 2024). Participants were randomly selected from the Population Information Registry, including 16,000 Finns aged 15–74 years in 2011; 7400 in 2015; 7800 in 2019 and 16,250 in 2023. Persons living in institutions, homeless individuals, expatriate Finns (including those living in Åland Islands), and persons whose mother tongue is other than Finnish, Swedish or Sámi were excluded from the study. The survey could be completed in either Finnish or Swedish.

A market research company, Taloustutkimus, collected the research data in 2011, whereas Statistics Finland was responsible for data collection in 2015, 2019 and 2023 (THL, 2024). In 2011–2019, the data were collected using a computer-assisted telephone interview (CATI). In 2023, the data (*n* = 16,250) collection method for the study changed from CATI to a combination of online and postal survey. However, to assess the change in methodology, a small part of the data were collected using CATI (*n* = 1250) and the rest with online and postal survey (*n* = 15,000). In the present study, we use combined data (*n* = 5977, response rate 36.9%), which includes responses to the online and postal surveys (*n* = 5349) and CATI (*n* = 628). The response rates varied across the years: 28.0% in 2011, 61.9% in 2015, 51.9% in 2019 and 36.9% in 2023. More detailed information about the methodology is included in the recently published statistical report (THL, 2024).

#### Demographics

*Information about gender* (women/men) *and age* was retrieved from the administrative population register. Age was categorised into five groups: 15–17 years, 18–29 years, 30–44 years, 45–59 years and 60–74 years.

#### Attitudes towards gambling

**Figure 1.**
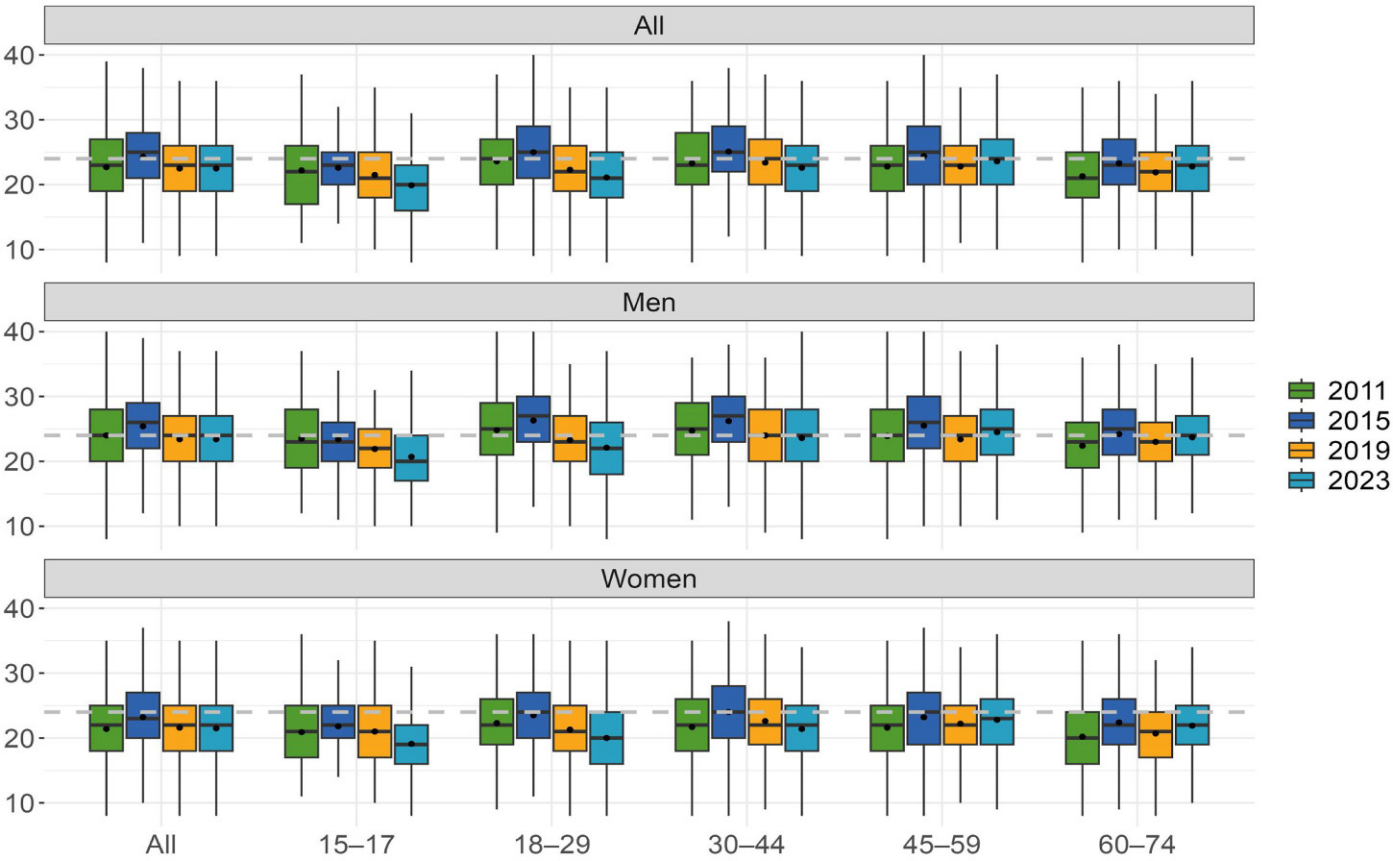
Attitudes towards gambling (ATGS-8) within genders and by age in 2011, 2015, 2019 and 2023.

We used the eight-item version of the Attitude Towards Gambling Scale (ATGS-8; Canale et al., 2016). This shorter version has been typically used in previous studies, instead of the original 14-item version (Kristensen et al., 2023). ATGS items are based on a Likert scale and include the following scoring: 1 = “strongly agree”, 2 = “agree”, 3 = “neither agree or disagree”, 4 = “disagree” and 5 = “strongly disagree”. A total ATGS-8 score (range 8–40) is formed by summing the scores of all items (Table 1): items 2, 3, 5 and 8 are reversely scored. The total scores are interpretated as follows: scores >24 indicate favourable (positive), a score of 24 indicates the overall neutral attitude, and scores below 24 indicate unfavourable attitudes towards gambling (negative) (Figure 1 and Table 2).

**Table 1.** Individual statements included in the Attitudes Towards Gambling Scale (ATGS-8), means, SD and proportions of respondents aged 15–74 in 2011, 2015, 2019 and 2023 (%).

	Year	Mean (SD)^1^	Strongly agree	Agree	Neither agree nor disagree	Disagree	Strongly disagree	I don't know/missing
1. People should have the right to gamble whenever they want								
	2023	3.4 (1.2)	20.9	36.6	12.8	22.6	6.7	0.4
	2019	3.5 (1.3)	21.2	40.0	8.7	21.2	8.4	0.5
	2015	3.6 (1.3)	29.7	36.6	6.5	18.9	7.8	0.3
	2011	3.2 (1.4)	20.6	31.8	6.4	23.9	16.3	1.1
2. There are too many opportunities for gambling nowadays*								
	2023	2.3 (1.1)	27.0	33.6	23.4	11.4	4.0	0.6
	2019	2.1 (1.2)	39.9	31.0	12.0	12.3	3.9	0.8
	2015	2.4 (1.3)	32.8	28.9	12.3	16.8	7.9	1.2
	2011	2.1 (1.2)	39.5	32.1	9.3	12.1	5.0	1.9
3. Gambling should be discouraged*								
	2023	1.5 (0.8)	64.3	25.5	6.8	1.7	1.2	0.4
	2019	1.4 (0.8)	69.8	21.6	4.4	2.8	1.0	0.5
	2015	1.6 (0.9)	60.7	25.2	6.6	5.1	1.8	0.5
	2011	1.4 (0.8)	71.0	20.2	3.8	2.7	1.6	0.7
4. Most people who gamble do so sensibly								
	2023	3.4 (1.0)	12.4	39.5	24.3	18.4	3.8	1.6
	2019	3.3 (1.2)	16.3	33.3	17.8	21.5	7.4	3.7
	2015	3.5 (1.2)	21.3	36.4	14.3	18.8	6.8	2.3
	2011	3.6 (1.3)	25.4	36.7	7.1	18.7	7.5	4.6
5. Gambling is dangerous for family life*								
	2023	2.5 (1.0)	15.9	38.7	24.6	17.3	2.9	0.6
	2019	2.4 (1.1)	18.8	42.5	17.3	16.2	4.0	1.2
	2015	2.6 (1.2)	16.0	38.5	15.7	22.7	6.1	1.0
	2011	2.4 (1.2)	22.5	41.2	12.0	16.4	4.9	3.1
6. On balance gambling is good for society								
	2023	2.5 (1.1)	2.7	17.5	27.4	33.3	18.5	0.6
	2019	2.8 (1.2)	5.8	27.7	18.9	30.3	16.0	1.7
	2015	3.2 (1.2)	10.9	35.1	19.5	23.8	9.3	1.4
	2011	2.8 (1.3)	8.7	28.6	14.8	26.6	17.3	4.0
7. Gambling livens up life								
	2023	3.0 (1.1)	4.4	36.1	27.0	22.2	9.8	0.6
	2019	3.0 (1.2)	5.5	39.5	19.3	21.4	13.0	1.3
	2015	3.2 (1.2)	9.0	44.5	15.5	19.1	10.9	1.0
	2011	3.1 (1.3)	9.0	41.9	12.3	19.5	14.3	3.0
8. It would be better if gambling was banned altogether*								
	2023	3.8 (1.2)	6.2	11.1	15.3	33.5	33.5	0.4
	2019	4.1 (1.1)	4.3	9.0	7.4	35.2	43.6	0.6
	2015	4.2 (1.1)	3.6	6.5	6.5	29.0	53.9	0.6
	2011	4.1 (1.2)	5.1	8.9	5.8	32.5	46.4	1.4

Weighted data based on gender, age and region of residence; The non-weighted data in 2011 (*n* = 4484), 2015 (*n* = 4515), 2019 (*n* = 3994) and 2023 (*n* = 5977); ^1^Scale: 1 = “strongly agree”, 2 = “agree”, 3 = neither agree nor disagree”, 4 = “disagree” and 5 = “strongly disagree”; *Four Attitudes Towards Gambling (ATGS-8) items have been reversely scored so that all item means >3.0 indicate an average attitude favourable to gambling and those below 3.0 indicate an average attitude unfavourable to gambling.

**Table 2.** Attitudes towards gambling by age within genders in 2011, 2015, 2019 and 2023 (means and SD).

ALL	2011 Mean (SD)	2015 Mean (SD)	2019 Mean (SD)	2023 Mean (SD)	*p*-value 2011 vs. 2023
	22.7 (5.6)	24.3 (5.5)	22.5 (5.2)	22.5 (5.4)	0.052
Age					
15−17 years	22.2 (5.8)	22.6 (5.1)	21.5 (4.7)	19.9 (5.3)	< .001
18−29 years	23.6 (5.4)	25.0 (5.3)	22.3 (5.0)	21.1 (5.5)	< .001
30−44 years	23.3 (5.5)	25.1 (5.5)	23.4 (5.2)	22.6 (5.6)	0.004
45−59 years	22.8 (5.5)	24.4 (5.8)	22.8 (5.3)	23.6 (5.3)	< .001
60−74 years	21.3 (5.5)	23.3 (5.4)	21.9 (5.3)	22.8 (5.1)	< .001
WOMEN	2011 Mean (SD)	2015 Mean (SD)	2019 Mean (SD)	2023 Mean (SD)	*p*-value 2011 vs. 2023
	21.4 (5.4)	23.2 (5.4)	21.6 (5.1)	21.5 (5.2)	0.584
Age					
15−17 years	20.9 (5.0)	21.8 (4.6)	21.0 (4.8)	19.1 (4.9)	0.018
18−29 years	22.3 (5.1)	23.5 (5.2)	21.3 (5.0)	20.0 (5.2)	< .001
30−44 years	21.8 (5.5)	24.0 (5.5)	22.6 (5.2)	21.4 (5.1)	0.305
45−59 years	21.6 (5.4)	23.2 (5.6)	22.2 (5.1)	22.8 (5.1)	< .001
60−74 years	20.2 (5.4)	22.4 (5.1)	20.7 (5.0)	21.9 (5.0)	< .001
MEN	2011 Mean (SD)	2015 Mean (SD)	2019 Mean (SD)	2023 Mean (SD)	*p*-value 2011 vs. 2023
	24.0 (5.4)	25.4 (5.5)	23.4 (5.2)	23.4 (5.5)	0.002
Age					
15−17 years	23.5 (6.1)	23.4 (5.5)	21.9 (4.5)	20.7 (5.5)	< .001
18−29 years	24.8 (5.4)	26.3 (5.1)	23.3 (4.7)	22.1 (5.6)	< .001
30−44 years	24.7 (5.1)	26.2 (5.2)	24.0 (5.2)	23.6 (5.7)	< .001
45−59 years	23.9 (5.2)	25.5 (5.7)	23.4 (5.2)	24.5 (5.4)	0.076
60−74 years	22.4 (5.5)	24.2 (5.1)	23.0 (5.4)	23.7 (5.1)	< .001

Significance (*p*) between time is determined by t-test for weighted data based on gender, age and region of residence; The non-weighted data in 2011 (*n* = 2367 females; *n* = 2117 males), 2015 (*n* = 2210 females; *n* = 2305 males), 2019 (*n* = 1964 females; *n* = 2030 males) and 2023 (*n* = 3150 females; *n* = 2827 males); The sum of eight Attitudes Towards Gambling (ATGS-8) items (a Likert scale: 1 = “strongly agree”, 2 = “agree”, 3 = “neither agree or disagree”, 4 = “disagree” and 5 = “strongly disagree”, 4 reversed items) forms a total ATGS-8 score (range 8−40) where a score of 24 represents the overall neutral attitude towards gambling, whereas scores >24 indicate favourable and those below 24 unfavourable attitudes.

Based on previous Finnish studies, the Cronbach's alpha value of the ATGS-8 has varied between 0.71 and 0.73 (Salonen et al., 2014, 2017). In 2023, the ATGS-8 reached the alpha value of 0.78. The original 14-item ATGS-instrument include two factors (Orford et al., 2009), whereas the factor analysis with the eight-item Finnish version has supported the use of two factors (Salonen et al., 2014). At that time, the item-total correlations varied from 0.28 to 0.51.

### Statistical analysis

Two data sets were combined and a new variable reflecting the year was created. The data were analysed with SPSS, version 29.0.2.0 (IBM Corp., Armonk, NY, USA). Furthermore, power calculations and the graphics of figure 1 were performed with the R programme, version 4.3.3. (R Core Team, 2023). Descriptive statistics included frequencies, percentages, means, SD, medians and quartiles. Statistical significance (*p*) of change between 2011 and 2023 was calculated using the independent samples *t*-test. All comparisons were performed across different age groups over time within women and men. The data were weighted to correspond to the target population of the study. In 2023 survey, the sum of sample weights thus corresponds to the target population of the study (*n* = 3,610,339; Statistics Finland's population data November 2023). In other words, each respondent represents an average of 604 people at the population level. The weighting criteria used were the age-gender distribution, regional distribution and urban-rural classification.

### Ethical considerations

Potential participants received written information about the study and the principles of voluntary participation. The Ethics committee of the Finnish Institute for Health and Welfare, Finland, approved the research protocols each year (2011: 6/2011§350–361; 2015: 10/2011§404–418; 2019: THL/774/6.02.01/2019; 2023: THL/1700/6.02.01/2023). The results of the study are presented in such a way that the respondents cannot be identified from of the results.

## Results

### Respondents

In 2011, 2015, 2019 and 2023, the number of women respondents aged 15–74 years was 2367 (mean ± SD = 44.5 ± 16.6 years), 2210 (45.6 ± 17.0 years), 1964 (46.5 ± 17.2 years) and 3150 (46.2 ± 17.2 years), respectively. The corresponding numbers for men were 2117 (43.8 ± 16.6 years), 2305 (44.8 ± 16.8 years), 2030 (45.5 ± 17.0 years) and 2827 (45.4 ± 17.1 years).

### Individual statements measuring attitudes

Table 1 shows means, SD and percentages for each of the attitudinal items included in the ATGS-8 instrument in 2011, 2015, 2019 and 2023. Three items out of eight (items 2, 3 and 5) produced mean scores that indicate unfavourable attitude towards gambling each year, whereas three other items (items 1, 4 and 8) produced mean scores that suggest favourable attitudes. The mean scores for the remaining two items (items 6 and 7) varied across years, reflecting sometimes unfavourable, sometimes neutral and sometimes favourable attitudes towards gambling. In each year, the item that indicated the most unfavourable attitude towards gambling was “Gambling should be discouraged” (item 3; mean: 1.4–1.6). Across the years, 85.9–91.4% of the respondents strongly agreed or agreed with this statement. Correspondingly, the item that indicated the most favourable attitudes towards gambling was “It would be better if gambling was banned altogether” (item 8; mean: 3.8–4.2). Across the years, 67.0–82.9% of the respondents strongly disagreed or disagreed with this item.

### Attitudes towards gambling by age within gender

Further analysis of the mean total ATGS-8 scores was performed by age group for men and women separately and for all respondents (Table 2). In addition, Figure 1 visualizes not only the mean and median ATGS-8 scores, but also quartiles. Overall, respondents’ attitudes towards gambling were unfavourable in each year. Mean scores ranged from 22.5 to 22.7. The only exception was 2015, when attitudes were slightly more favourable (mean: 24.3). There was no statistically significant change in attitudes from 2011 to 2023. Women's attitudes have been unfavourable every year (mean: 21.4–23.2), whereas men's attitudes have varied from year to year. Men's attitudes were initially neutral in 2011 (mean: 24.0) before turning favourable in 2015 (mean 25.4). Since 2019, men's attitudes towards gambling have been unfavourable, with an average score of 23.4.

From 2011 to 2023, the mean attitude scores towards gambling were generally unfavourable across all age groups, with the exception of 2015 (Table 2). In 2015, those aged 18–59 years exhibited favourable attitudes, with mean scores ranging from 24.4 to 25.1. Over the same period, although attitudes towards gambling became more favourable among those aged 45–74 years, they remained negative overall, whereas other age groups developed increasingly unfavourable attitudes. Among women, attitudes towards gambling were unfavourable across all age groups except in 2015, when women aged 30–44 years had neutral attitudes. From 2011 to 2023, attitudes among women aged 15–29 years became more unfavourable, whereas those aged 30–44 years remained unchanged. By contrast, attitudes of women aged 45–74 years became more favourable. For men aged 15–44 years, attitudes towards gambling became more unfavourable from 2011 to 2023, whereas they became more favourable for those aged 60–74 years. The 45–59 years age group showed no change in attitudes and was the only group with favourable attitudes in 2023.

## Discussion

The present study has investigated how attitudes towards gambling have changed from 2011 to 2023 in Finland, analysing differences by gender and age using nationally representative population samples from four cross-sectional survey studies. Based on the results, women's attitudes were unfavourable each year, whereas men's attitudes have varied from year to year. Among both men and women, attitudes became more unfavourable in younger age groups, whereas attitudes became more favourable in older age groups. In 2023, men aged 45–59 years were the only age group with favourable attitudes.

### Finns’ overall attitudes towards gambling were mainly negative

Overall, Finns’ attitudes towards gambling were mainly unfavourable, and no significant changes have occurred between 2011 and 2023. Women's attitudes were unfavourable each year, whereas men's attitudes fluctuated over time. Attitudes among those aged 45–74 years became slightly more favourable, remaining still negative overall. By contrast, attitudes among younger age groups became more unfavourable during the same period.

This study found that the general attitudes towards gambling were unfavourable in most years. The result was relatively similar compared to other previous studies internationally (Kristensen et al., 2023). Recent research suggests that public attitudes towards gambling are increasingly unfavorable, reflecting a broader global shift. Orford (2019) describes this trend as a “backlash against commercial gambling”, highlighting a growing societal resistance to the industry's expansion and its perceived negative impacts. This shift is evident in various regions where public awareness of gambling-related harms has intensified, alongside a rise in regulatory measures aimed at curbing these impacts. Recent examples, such as stricter advertising restrictions and the implementation of more robust responsible gambling measures, underscore this movement. These actions illustrate a societal response that aligns with our observed attitudinal trends, supporting the notion that public perception is becoming increasingly critical of commercial gambling practices.

Extensive research has indicated a close link between attitudes and behaviour (Glasman & Albarracín, 2006). According to the theory of planned behaviour (Ajzen, 1991; 2012), behaviours such as gambling can be predicted based on an individual's attitudes, subjective norms and perceived control over the behaviour (Martin et al., 2010; Sussman & Gifford, 2019). From this theoretical perspective, it is somewhat surprising that no significant changes in the overall attitudes were found between 2011 and 2023 even though the prevalence of past-year gambling in Finland decreased from 78% to 70% during this period (THL, 2024). Moreover, previous studies have shown that favourable attitudes towards gambling are associated with higher gambling intensity, such as gambling more frequently and spending much money on gambling (Kristensen et al., 2023). Generally, gambling in Finland has become more occasional: in 2011, the most common gambling frequency was once a week, whereas, in 2023, gambling typically occurred less than once a month (THL, 2024; Grönroos et al., 2024). This discrepancy between behaviour and attitudes has been observed not only for gambling, but also for substance use problems (Fiedor & Seidlová, 2022). Because a large portion of the population already holds negative views about gambling, those attitudes might not shift significantly, even if actual gambling behaviour changes. The decrease in gambling may be also driven by external factors such as economic constraints or accessibility, whereas attitudes remain stable because they reflect deeper cultural or personal values that do not change quickly in response to external changes. This downward trend in gambling intensity and at-risk gambling may reflect stricter harm prevention methods implemented in the early 2020s, such as mandatory identification and the reduction in the number of EGMs (Russell et al., 2023).

Another key finding was that although the general attitudes towards gambling have been unfavourable every year, the year 2015 appears to be an exception compared to other years, with attitudes being favourable. At the time, men's attitudes were favourable across all age groups except for minors. Additionally, women aged 30–44 years had neutral attitudes, although, in all other years and across all age groups, women's attitudes were unfavourable. This may be partly because, in 2015, public debate on gambling and gambling companies was less critical and active compared to recent years. This shift in discourse may have influenced Finn's attitudes towards gambling at the time of data collection for the most recent two survey studies in 2019 and in 2023. Furthermore, as noted by Macey et al. (2023), around the same time, stories from individuals with personal experiences of gambling problems have become more prominent in public discourse. As a result, gambling-related harms have been more visible, which may have contributed to a shift in people's attitudes, making them more unfavourable towards gambling. The public interest in the Finnish gambling system, along with criticism of its justifications, grew significantly from the mid-2010s onward, culminating in 2019 when Veikkaus’ advertising campaign was heavily criticized in the media (Kankainen, 2024). As politicians or beneficiaries increasingly distanced themselves from the traditional gambling system and public criticisms emerged, it is likely that Finnish citizens became more aware of gambling-related issues and reported more unfavourable attitudes towards gambling compared to a decade earlier. Finally, the long-term efforts in Finland to prevent and reduce gambling-related harm, along with the development of treatment and help for gambling problems may have influenced attitudes towards gambling.

### Opposite trends in attitudes among the youngest and oldest

Among women, attitudes became more unfavourable for those aged 15–29 years, whereas they became more favourable for those aged 45–74 years. Among men, attitudes became more unfavourable for those aged 15–44 years, whereas they became more favourable for those aged 60–74 years. The results concerning gender differences were also in line with the previous literature: women's attitudes towards gambling have been more unfavourable than men in every year (Orford et al., 2009; Salonen et al., 2014, 2017; Donaldson et al., 2016; Fiedor et al., 2019; Pallesen et al., 2020). Gambling is notably more popular among men than women, which may partly explain men's more positive attitudes (Abbott et al., 2018). Furthermore, a possible explanation for this could be that men are in general more prone to risk-taking. For example, a study by Wong et al. (2013) found that risk-taking and impulsive behaviour mediated gender differences in gambling engagement and problem gambling. Another possible explanation could be that women are more often indirectly affected by close person's gambling problems which probably contributes to their attitudes towards gambling (Castrén et al., 2021). According to the latest Finnish Gambling survey (THL, 2024), the proportion of women with a family member who had problematic gambling was higher than the corresponding proportion among men in 2023. However, when examining those with a friend who had problematic gambling, the situation was reversed: the proportion of men was higher than the corresponding proportion among women.

Another interesting finding was that women's attitudes remained unfavourable every year, whereas men's attitudes varied slightly. This could indicate that men's attitudes are more susceptible to changes in external influences such as societal trends, media, or policy shifts, whereas women's attitudes may be more stable or resistant to such factors. Women may have more consistent negative views on gambling as a result of cultural expectations discouraging financial risks, whereas gambling is seen as more socially acceptable for men, leading to greater variability. Furthermore, men also engage in gambling more frequently, which could lead to shifting attitudes based on changes in gambling trends. In 2011, young men's (aged 18–29 years) attitudes were slightly positive, whereas men aged 45 years or older had more critical attitude. In 2023, the attitudes of young men and men aged 45 years or older have shifted to opposite directions: younger men have adopted more critical attitude. This finding is in contradiction to previous findings that have found an inverse relationship between positive attitude and age (Kristensen et al., 2023). There could be several explanations for this. Younger generations might have better access to information, and therefore be more aware of the risks associated with gambling. Older men may have a more traditional view of gambling as a form of entertainment, especially if they grew up during a time when gambling was more socially accepted and not as heavily scrutinized. Younger men, however, might associate gambling more with online platforms, microtransactions and aggressive marketing, which can contribute to a more negative perspective. Overall, game type preferences and harm potential of games among different age groups might have an influence on attitudes.

Lastly, the present study has described the situation and trends before the shift from a monopoly system to a licensing system, which will occur in January 2026 or after. The justification for this reform is written in the Government Programme: “The aim of the reform is to prevent and reduce economic, social and health-related harm resulting from gambling and to improve the channelling rate of the gambling system” (The Finnish Government, 2023). However, the potential effects of this reform not only on gambling and related harm, but also on public attitudes towards gambling need to be closely monitored. It is likely, that this shift will lead to increased advertising for online gambling because of the competition between operators, which in turn might contribute to a potential increase in unfavourable gambling behaviour and impact attitudes towards gambling.

### Strengths and limitations

The methodology of these four data sets has many similarities and strengths, but also some differences. Differences worth mentioning include how the survey was introduced to the participants, who collected the data and how the data were collected. Partly because of these choices, the response rates of the Finnish Gambling surveys varied years from 28.0% to 61.9%. The response rate dropped between 2019 to 2023 from 51.9% to 36.9%. However, non-response analyses of the basic report indicate that the 2023 data were still representing the Finnish population (THL, 2024). Different response activity might influence to the comparability of the results between years. The reason for the decrease in response rate in 2023 was a mode change from CATI to a combination of online and postal survey. However, to assess the impact of this mode change, some of the data were still collected using CATI in 2023, and a response rate for CATI part was 50.7% (THL, 2024). Furthermore, the basic analysis showed that there was no mode difference in attitudes towards gambling. Based on further analyses with 2015 data, socio-economic status was linked with lower response rate, which may cause bias (Kontto et al., 2020). This is important to consider because low socio-economic status has been linked with more positive attitudes towards gambling (Salonen et al., 2014). Previously validated ATGS-8 has been widely used around the world (Kristensen et al., 2023) and considered as a reliable measure for attitudes towards gambling not only among both gamblers, but also among those who do not gamble (Canale et al., 2016). Furthermore, the sizes of different sub-groups were relatively small, particularly among those aged 15–17 years.

A further limitation of this study is that attitudes towards gambling were analysed based on age and gender. Future research should adopt a broader approach by examining various micro-level factors that influence attitudes, such as gambling behaviour, political orientation, trust in authority, and religious affiliation. While previous studies on the relationship between political orientation, trust in authority, and attitudes towards gambling are scarce, the association between religious orientation and gambling attitudes has been studied more extensively (e.g., McAllister, 2014; Gavriel-Fried, 2015; Sanscartier et al., 2020). In this study, measures of political orientation, trust in authority, and religious affiliation were not included due to the absence of relevant variables in our data set. In future studies, it would also be valuable to consider macro-level factors, such as the political and ideological climate, public discourse on gambling, and the general overall economic situation.

It is noteworthy that attitudes towards gambling were inquired about on a general level, not by specific game types (e.g. lottery games or betting games). In the survey questionnaire, a brief definition of gambling was provided right before the ATGS-8 questions: “Gambling refers to playing games for example lottery games, slot machines, scratch cards, sports and horse games, betting games which are also available online”. Despite this, respondents may have considered specific types of gambling when answering the questions, which may have influenced the responses.

## Conclusions

To our knowledge, research on attitudes towards gambling within gender is scarce. This study contributed to filling this gap by examining how attitudes towards gambling have changed from 2011 to 2023 in Finland among women and men by age using nationally representative population survey. In general, the attitudes of Finns towards gambling were mainly unfavourable, with no significant changes observed from 2011 to 2023. The only exception was 2015, when attitudes were favourable. As noted in the earlier study (Salonen et al., 2017), attitudes became significantly more favourable in Finland from 2011 to 2015. However, in 2019 and 2023, attitudes no longer shifted in a more favourable direction. Women's attitudes were unfavourable throughout the years, whereas men's attitudes fluctuated over time. Among both men and women, attitudes became more unfavourable in younger age groups, whereas attitudes became more favourable in older age groups. In 2023, men aged 45–59 years were the only age group with favourable attitudes. Finland is set to replace its gambling monopoly with a licensing system in the near future, opening the online gambling market to competition. The shift to a licensing system and the changes it causes (e.g. in marketing) make it important to monitor more closely in the coming years whether there will be a change in public attitudes.
